# Resection of Cesarean Scar Pregnancy at Six Weeks of Gestation with Laminaria Cervical Dilatation under Sonographic and Hysteroscopic Guidance

**DOI:** 10.1155/2015/685761

**Published:** 2015-09-15

**Authors:** Tatsuji Hoshino, Taito Miyamoto, Shinya Yoshioka

**Affiliations:** Department of Obstetrics and Gynecology, Kobe City Medical Center General Hospital, Minami-Machi 2-1-1, Chuo-Ward, Kobe 650-0047, Japan

## Abstract

In cases of fetal heartbeat- (FHB-) positive cesarean scar pregnancy (CSP), the embryo and placenta grow rapidly week by week. We experienced an FHB-positive CSP case at 6 weeks of gestation and assessed the CSP in detail with transvaginal ultrasound and transabdominal ultrasound (TAUS), preoperatively. We performed Laminaria cervical dilatation under TAUS guidance and performed hysteroscopic resection of the pregnancy conceptus and curettage under hysteroscopic and TAUS guidance. We identified the gestational sac attached to the cesarean scar pouch with small plane, decidua basalis, and chorionic villi and present the clinical history and other findings. We also reviewed the related literature and found 76 previous studies, with six cases of FHB-positive CSP that contained hysteroscopic color images of the CSP. We present a review of selected cases. The implantation site was the anterior wall in almost all cases. Cervical dilatation was mainly performed using a Hegar dilator; ours was the only case using Laminaria dilatation. Transcervical resections were performed mainly under ultrasound guidance, with only one case undergoing laparoscopy. Electrocoagulation was performed in three of the six cases.

## 1. Introduction

In cases of fetal heartbeat- (FHB-) positive cesarean scar pregnancy (CSP), resecting the pregnancy contents as early as possible is recommended to avoid catastrophic outcomes such as uterine rupture or massive bleeding.

We experienced an FHB-positive CSP case at 6 weeks of gestation and were able to assess the CSP in detail using transvaginal ultrasound (TVUS) and transabdominal ultrasound (TAUS), preoperatively. We performed Laminaria cervical dilatation under TVUS and TAUS guidance to adequately visualize the CSP and to more easily resect the pregnancy contents. We also performed hysteroscopic resection of the pregnancy conceptus and curettage under TAUS guidance. We acquired images that demonstrated the gestational sac (GS) attached to the maternal uterus, decidua basalis, and chorionic villi with small plane. We discuss the clinical history and findings in this case. We also reviewed the related literature and found 76 previous studies, with six cases of FHB-positive CSP that contained hysteroscopic color images of the CSP. We present a review of selected cases.

## 2. Case Report

**Figure 1:**
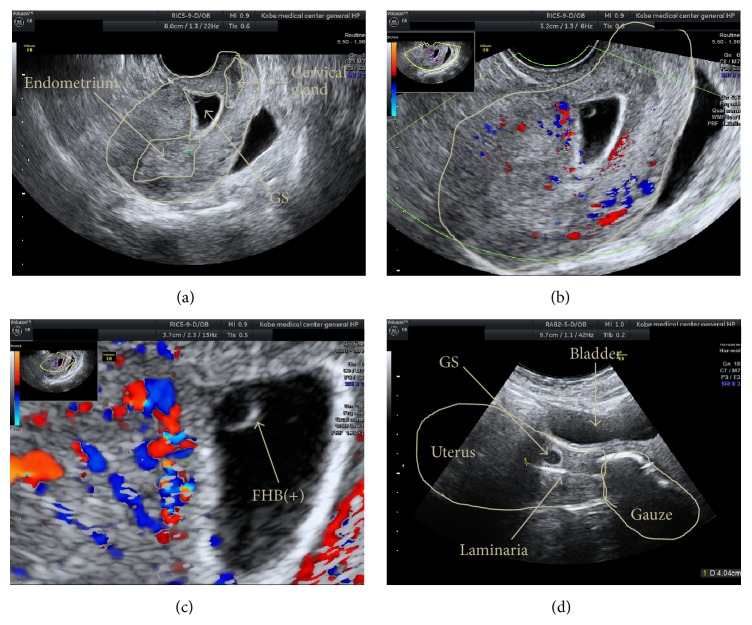
Ultrasonographic findings in cesarean scar pregnancy. (a) The gestational sac (GS) was located in the cesarean scar pouch. The uterine cavity and cervical glands were empty. (b) The GS was triangular in shape inside the cesarean scar pouch. (c) Fetal heartbeat was positive in the hyperechoic portion of the Yolk Sac, and the fundal side of the triangular-shaped GS was rich in blood supply. (d) The GS shape changed to round after insertion of the Laminaria tent.

The patient was a 28-year-old female who had one miscarriage history and one cesarean section history for twin pregnancy at 36 weeks of gestation. She had been amenorrheal since her last menstrual period. Her menstrual cycle was 27 days and regular, and she was introduced to our hospital because of suspicion of CSP. She was presumed to be 6 w 0 d of gestation based on the date of her last menstrual period. TVUS showed that her cesarean scar defect site (CSDS) had enlarged to a triangular shape, with the presence of an 18.7 mm GS containing an FHB and yolk sac (Figures 1(a) and 1(b)).

Color Doppler ultrasound (Voluson E8, GE Healthcare, Milwaukee, WI, USA) detected blood flow in the anterior-fundal side of the space; therefore, the decidua basalis and chorion frondosum were presumed to be present on that side (Figure 1(c)). Blood human chorionic gonadotropin (hCG) level was 21,235 IU/L. After providing a full explanation and obtaining informed consent, the patient was admitted for hysteroscopic resection of pregnancy contents under TAUS guidance after sufficient cervical dilatation following two Laminaria insertions.

**Figure 2:**
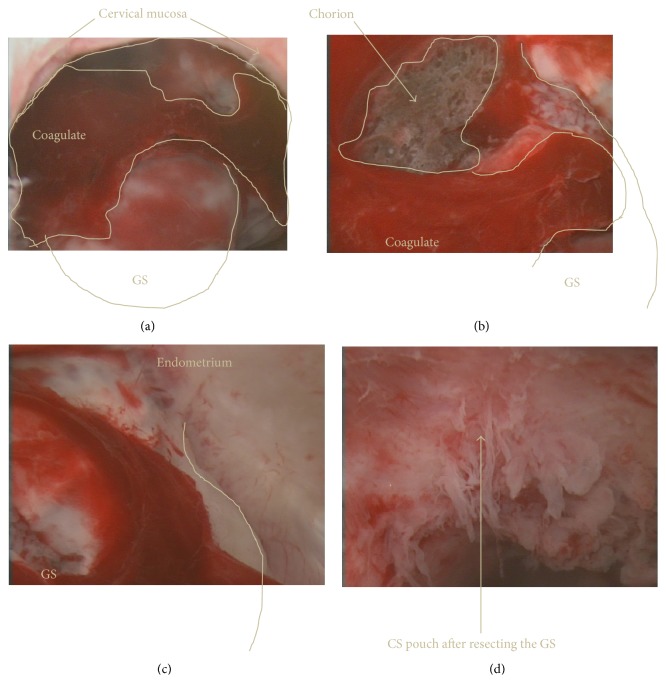
Hysteroscopic findings in cesarean scar pregnancy. (a) The round-shaped gestational sac (GS) was located in the cesarean scar pouch just beyond the cervical canal. The GS was partly covered with clotted blood. (b) The GS was attached to the cesarean scar pouch with chorionic tissue. (c) The GS was surrounded with thick endometrial glands. (d) The GS was resected in the cesarean scar pouch.

We carefully inserted one Laminaria tent along the posterior wall to avoid injury to the GS, using TAUS guidance. After the first single Laminaria tent insertion, the GS transformed from triangular in shape to more oval in shape, and the anterior wall thickness in the CSDS increased to 6 mm (Figure 1(d)). The next day, the second Laminaria cervical dilatation was performed. At this point, TAUS detected a triangular-shaped GS, yolk sac, and FHB. Laminaria insertion was repeated and the first Laminaria was inserted into the cervical canal along the posterior wall like yesterday so as to avoid injury to the pregnancy contents. The second Laminaria was then inserted into the cervical canal along the posterior side of the first Laminaria, also to avoid injury to the pregnancy contents. A third Laminaria was then inserted into the cervical canal along the posterior side of the first two Laminaria tents, which were positioned side by side in the horizontal plane. After inserting a total of four M-sized Laminaria tents, two half-sized gauzes were inserted into the vagina near the cervix and sufficient chlorhexidine liquid was added. TAUS and TVUS detected a round-shaped GS and identified that the Laminaria tents had not perforated the uterine wall. Under general anesthesia, to provide the best TAUS view, we first inserted an indwelling balloon catheter into the bladder and injected 250 mL physiological saline solution. Next, we assessed the CSP hysteroscopically under TAUS guidance, which showed that the cervical canal and lower part of the uterine cavity were sufficiently enlarged following the Laminaria cervical dilatation, and the GS, which was covered with the decidua capsularis and chorion laeve, implantation site, and chorion frondosum, were clearly observed (Figures 2(a)–2(c)). The GS was implanted in the anterior wall of the triangular-shaped CSPD, from ten to two o'clock on the anterior-fundal side. No large supplying blood vessel was observed. We attempted to bluntly resect the GS with the chorion from the CSDS mucosa with a u-shaped wire loop electrode, but the electrode could not reach the border. The pregnancy contents were then resected with placental forceps under TAUS guidance, and the CSDS was curetted with an appropriately sized curette. Electrocoagulation was not performed to avoid burning injury and almost no bleeding was observed from the implantation site (Figure 2(d)). Intraoperative blood loss was minimal and not countable. The extraction specimen was chorion and decidua macroscopically, but no fetus was detected. Postoperative pathological examination also identified chorion and decidua, but no fetus was identified microscopically.

On the first postoperative day, the patient was in good condition and had no fever. Blood laboratory examination revealed: C-reactive protein level, 0.55 mg/dL (range 0.00–0.50 mg/dL); white blood cell count, 14.1 × 10^3^/*μ*L (range 3.9–9.8 × 10^3^/*μ*L); hematocrit, 38.9% (range 33.5–45.1%); hemoglobin, 13.5 g/dL (range 11.1–15.1 g/dL); and platelets, 22.1 × 10^4^/*μ*L (range 13–37 × 10^4^/*μ*L). Preoperative laboratory blood examination results 2 days previously revealed C-reactive protein, 0.04 mg/dL; white blood cell count, 9.1 × 10^3^/*μ*L; hemoglobin, 14.3 g/dL; hematocrit, 41.6%; and platelets, 21.5 × 10^4^/*μ*L. Physical examination identified a small amount of brownish vaginal discharge and no other significant findings. TVUS showed no significant findings. The endometrial mucosa was 16.8 mm thick because endometrial mucosal curettage was not performed. On the second postoperative day, the patient was discharged from the hospital and was recommended to follow-up in the out-patient department for hCG level measurement and body temperature. TVUS was performed 12 days postoperatively (her first visit after discharge), and no significant findings were detected. Her hCG level at this visit was 65.2 mIU/L. The next visit occurred 4 weeks later and her hCG level was then 1.3 (nonpregnant level < 5.0 mIU/mL). Her menstruation began approximately 3 weeks after the CSP resection.

## 3. Discussion and Literature Review

We reviewed the chapters on CSP in Williams Obstetrics, 24th edition [1], and UpToDate [2] and each of the references in these chapters, which showed that articles concerning hysteroscopic CSP resection were reported by Wang et al. [3, 4] in the English literature. Therefore, we searched the MEDLINE/PubMed database using the terms “(Wang CJ OR Hysteroscopy OR Resectoscope) AND Cesarean scar pregnancy” to identify FHB-positive cases in articles that included hysteroscopic figures of CSP up to 2015.

We identified 76 articles and then excluded Russian, Chinese, and French articles and inaccessible articles (14 articles). The remaining 62 articles with color figures were examined as original articles in detail and, from these, we identified six FHB-positive CSP cases including ours. Most were managed with hysteroscopic resection at the time of FHB-positive detection (Table 1) [3–7]. Patients' ages ranged from 29 to 44 years with a mean of 33.5 years. The number of previous lower segment cesarean sections was one in three cases and two in three cases. The gestational weeks were 5 weeks' gestation in one case, 6 weeks in one case, and 7 weeks in four cases. The diagnosis was performed by TVUS and/or TAUS and no case described that the diagnosis was performed by magnetic resonance imaging (MRI). Beta-hCG level ranged from 11,082 to 99,554 mIU/mL. The implantation site was the anterior wall in almost all cases and no posterior wall implantation was reported. Cervical dilatation was performed primarily by Hegar dilator; ours was the only case using Laminaria dilatation. Transcervical resections were performed mainly under US guidance, and only one case underwent laparoscopy. Electrocoagulation was performed in three of the six cases.

## 4. Considerations

### 4.1. Regarding CSP

In CSP, chorion may invade neighboring organs such as the bladder, which could lead to catastrophic outcomes including massive intraperitoneal bleeding, uterine rupture, or maternal death. Treatment is also more difficult when delayed [8, 9]. The ideal treatment for CSP involves an early diagnosis and resection of the pregnancy contents as soon as possible. It is ideal to resect the pregnancy contents by minimally invasive therapy without opening the abdominal wall or uterine wall, and without prescribing toxic agents such as methotrexate [10]. For maternal health reasons, it is better to plan for a healthy live baby in a future pregnancy than allow a CSP to progress. If the CSP is allowed to progress, the pregnancy contents increase in size and chorion invades the uterine wall or neighboring organs. This may create a need for hysterectomy and eliminate the possibility of any fertility-preserving therapy.

### 4.2. Regarding the Case

#### 4.2.1. The Usefulness of Hysteroscopic Resection as a Treatment

After fully and directly observing the pregnancy contents with hysteroscopy, it is considered relatively easy to resect the pregnancy contents under TAUS guidance and to observe the CSDS after resection to confirm that there is no bleeding or residual tissue present and no uterine wall injury. It is best to avoid electrocoagulation if possible because this method increases scar organization and can leave a tissue deficit.

#### 4.2.2. The Usefulness of Laminaria Cervical Dilatation

Using Laminaria cervical dilatation, the endocervical canal is expanded slowly and largely without cervical laceration contrary to using Hegar cervical dilatation. Repeating the cervical Laminaria dilatation several times expands the cervical canal to a greater size. Also, because the cervical canal and lower uterine segment are dilated more fully, the cesarean scar pouch, GS, and surrounding structures can be easily observed by hysteroscope, and the decidua vera, chorion laeve, and chorion frondosum and supplying vessels are also easily seen. This permits resection of the pregnancy contents easily and safely under TAUS guidance.

Insertion of the Laminaria requires careful manipulation and it is important not to injure the GS, decidua, chorion, and supplying vessels and to avoid bleeding during insertion. According to the cases we reviewed, there was no case except ours in which Laminaria cervical dilatation was performed. Laminaria tents are available in various sizes from ultra-thin to very thick, offer good operability, and are made of a strong material, which are useful characteristics.

#### 4.2.3. Regarding the Embryo and Placenta at 6 Weeks' Gestation

The embryo at 6 weeks' gestation is equivalent to 8 and 9 of the Carnegie stages in human embryology. In Takemura's standard figures of TVUS findings during gestation, the embryonic size (crown-rump length) ranges from 4 to 8 mm at 6 weeks and the size of the GS ranges from 18 to 29 mm [9]. Ontogenesis is ongoing. The uterine cavity space still remains and the decidua capsularis and decidua parietalis are not united. Because the uterine cavity is expanded by fluid pressure during hysteroscopic observation, the GS can be identified surrounded by decidua capsularis and chorionic villi. However, because the GS is surrounded by opaque decidua capsularis and chorionic villi, an embryo and yolk sac are not observable by hysteroscopy.

### 4.3. Bibliographic Considerations

#### 4.3.1. FHB-Positive Cases

At 5 weeks' gestation, FHB is usually identifiable, which allows for a presumption of gestational weeks based on the size of the GS and crown-rump length. When FHB is negative, gestational weeks are presumed from the last menses or cessation of fetal growth is considered, and the exact gestational weeks can be difficult to determine.

#### 4.3.2. Regarding Choosing Articles Containing Hysteroscopic Images

CSP imaging information has increased in recent years, including MRI, TAUS, TVUS, and color Doppler imaging. Macroscopic image findings have also become more abundant. A color photograph of the transverse section of a case of CSP has been published in Williams Obstetrics [1], and we previously published a color photograph of the cut surface of the uterus and pregnancy contents in CSP in the journal of the International Journal of Obstetrics and Gynecology [11]. Because MRI can image target organs, surrounding organs, and the entire body, it is a very useful modality; however, it is difficult to obtain images frequently and repeatedly such as at the time of pelvic examination, Laminaria insertion, and intraoperatively in CSP. Although ultrasound resolution has improved and clear photographs can now be obtained, images have not been as clear as macroscopic and hysteroscopic images until recently. We selected articles containing hysteroscopic color images of CSP that most accurately showed the CSP. We also selected articles in which primary CSP resection was performed after hysteroscopic observation, which is a direct observation technique that can assess the implantation site* in situ* preoperatively.

## 5. Conclusions

The cervical canal can be dilated safely with careful and gentle Laminaria tent insertion in cases of CSP at 6 weeks of gestation. Clear hysteroscopic observation of the GS, chorion, decidua, and the border was possible after dilatation of the cervical canal and lower uterine segment because the blind areas were reduced. Hysteroscopic resection of CSP contents was safely performed using TAUS. Direct hysteroscopic observation revealed no residual mass in the cesarean scar pouch or the uterine cavity. Four successful cases of CSP resection at 7 weeks of gestation are included in this literature review, suggesting that primary hysteroscopic resection of FHB-positive CSP up to 7 weeks can be performed safely in cases using electrocoagulation, postoperative gauze packing, and hemostatic agents.

## Figures and Tables

**Table 1:** Primary hysteroscopic resection of fetal heartbeat-positive cesarean scar pregnancy cases that appeared in journals containing hysteroscopic color figures.

Case	Authors, journal or congress, year	Age (y) gravity and parity previous CS (*n*)	Mode of conception	Bleeding	Gestation (weeks and days or weeks)	FHB	Size of GS, CRL	Diagnostic modalities (TVUS, TAUS, and MRI)	Preoperative beta-hCG level (mIU/mL)	Implantation site	Cervical dilatation (Laminaria or Hegar)	Management	TAUS guidance or laparoscopic assist	Remarks
1	Hoshino T et al., ISUOG, 2014	29 G2P1 1	Spontaneous	No	6 w 1 d	+	GS: 18.7 mm	TVUS, TAUS	21,235	Anterior wall, fundal side	Laminaria	Hysteroscopic resection	TAUS	

2	Chang et al., Fertil Steril, 2011 [5]	29 G3P2 2	Not documented	Vaginal bleeding	7 weeks	+	GS: 21 mm CRL: 8.1 mm	TVUS	51,775	Anterior wall, right fundal side	Hegar dilatation to 11 mm	Diluted vasopressin injection, resection with wire loop electrode	Not documented	

3	Wang et al., Fertil Steril, 2010 [3]	31 G3P1 1	IVF, 4 embryos transferred	Vaginal bleeding	7 weeks	+	GS: + CRL: +	TVUS, TAUS	99,544	Not documented	Hegar dilatation to 11 mm	Hysteroscopic-directed evacuation and D&C	Under US control	Heterotopic CSP. CS at 39 weeks, live male 3250 g

4	Robinson et al., Fertil Steril, 2009 [6]	44 G6P2 2	Spontaneous	No	5 w 6 d	+	GS: 7.5 mm CRL: 1.3 mm	Follow-up ultrasound	11,082	Anterior wall, fundal side	Not needed	Laparoscope-assisted hysteroscopy	Laparoscopy assist	

5	Deans and Abbott, Fertil Steril, 2010 [7]	41 Not documented 1	Not documented	Not documented	7 weeks	+	Not documented	Not documented	78,000	Not documented	Not documented	Hysteroscopy	Not documented	

6	Wang et al., BJOG, 2005 [4]	36 G5P2 2	Spontaneous	Not documented	7 w 3 d	+	Not documented	TVUS	28,338	Anterior wall	Hegar dilatation to 12 mm	Operative hysteroscopy accompanied by suction curettage	Not documented	

Note: y: years old; CS: cesarean section; *n*: number; hCG: human chorionic gonadotropin; FHB: fetal heartbeat; GS: gestational sac; CRL: crown-rump length; TVUS: transvaginal ultrasound; TAUS: transabdominal ultrasound; MRI: magnetic resonance imaging; G: gravidity; P: parity; D&C: dilatation and curettage; CSP: cesarean scar pregnancy.
